# Long-Term Real-World Outcomes and Insights of Biologic Therapies in Chronic Rhinosinusitis with Nasal Polyps

**DOI:** 10.3390/ijms26104694

**Published:** 2025-05-14

**Authors:** Reut Book, Anna Lazutkin, Ron Eliashar

**Affiliations:** Department of Otolaryngology/Head and Neck Surgery, Hadassah Medical Center, Faculty of Medicine, Hebrew University of Jerusalem, Jerusalem 9112102, Israel

**Keywords:** chronic rhinosinusitis, biologic therapy, dupilumab, nasal polyps, EPOS, EUFOREA, SNOT-22, long-term follow-up, injection interval, olfaction, quality of life

## Abstract

Chronic rhinosinusitis with nasal polyps is a Type 2 inflammatory disease associated with a significant burden on quality of life. While biological therapies have shown efficacy in randomized controlled trials, data on long-term real-world outcomes remain limited. This retrospective cohort study evaluated the clinical efficacy, safety, and treatment dynamics of biologics, particularly anti-IL-4 (dupilumab), over a five-year period at a tertiary medical center. Fifty-two patients with CRSwNP meeting the EPOS/EUFOREA eligibility criteria were included. Clinical parameters, including nasal polyp score, SNOT-22, and olfactory function, were assessed across follow-up intervals. Anti-IL-4 therapy demonstrated the most consistent and sustained improvements in all clinical parameters, with a significant proportion of patients maintaining response beyond 36 months. A subset of patients underwent interval extension of dupilumab injections without loss of efficacy. Subdomain analysis of the SNOT-22 questionnaire revealed improvements predominantly in nasal and emotional domains. Treatment response, assessed according to the EUFOREA criteria, favored anti-IL-4 over anti-IL-5 and anti-IgE. Side effects were infrequent and mostly mild. These findings support the durable effectiveness of biologics in real-world CRSwNP management and suggest that tapering down the injection intervals may be a feasible strategy for selected patients. Further studies are needed to refine treatment response definitions and optimize patient-specific therapeutic approaches.

## 1. Introduction

Chronic rhinosinusitis (CRS) is an inflammatory condition of the nose and sinuses, characterized by nasal obstruction or discharge, facial pain or pressure, and reduction or loss of smell for ≥12 weeks. CRS is generally divided into CRS with or without nasal polyposis (CRSwNP and CRSsNP, respectively) [1].

CRSwNP has a significant negative impact on the patient’s quality of life. Affected patients often present with other comorbidities, such as asthma, allergic rhinitis, and intolerance to non-steroidal anti-inflammatory drugs (NSAIDs), contributing to the severity of the disease [1,2,3]. Despite surgery [Endoscopic Sinus Surgery (ESS)] and ongoing intranasal corticosteroid (INCS) and/or systemic corticosteroid therapy, the estimated number of patients remaining symptomatic with incomplete disease control is high, verging around 40–50% of cases [4,5].

CRSwNP is mainly classified as a Type 2 inflammatory disease [1]. As such, it is driven by the key cytokines Interleukin (IL)-4, IL-5, and IL-13^5^. In addition, IgE is also thought to play a central role in CRSwNP pathogenesis by activating and generating degranulation of mast cells and basophils, thus causing activation of other immune cells, including Th2 cells, and eosinophils [6].

Biological treatments can offer a more targeted approach by blocking specific cytokines and immune pathways involved in Type 2 inflammation. These therapies have transformed the treatment landscape for patients with severe CRSwNP who are unresponsive to conventional treatments. Biologics such as anti-IgE (omalizumab), anti-IL-5 (mepolizumab or benralizumab), and anti-IL-4Rα (dupilumab), have shown significant efficacy in reducing polyp size, improving symptoms, and reducing the need for corticosteroids or surgery [7,8,9].

While randomized controlled trials (RCTs) and real-world studies have firmly established the efficacy and safety of those biological treatments in the management of CRSwNP, the evidence is mostly for the first year of treatment. Very few studies have published data on long-term follow-ups. This real-world retrospective cohort study evaluates the long-term therapeutic efficacy and safety of different biological treatments and provides more evidence for the sustained efficacy of treatment while increasing the injection intervals for anti-IL-4 injections.

## 2. Results

### 2.1. Patient’s Characteristics

Fifty-two patients were included in this study. All patients met the EPOS/EUFOREA criteria for biologic eligibility.

The patients were followed for up to 5 years, over 175 clinic visits, with follow-ups ranging from 1 to 19 visits per patient. The patient’s baseline demographics and characteristics are presented in Table 1.

### 2.2. Treatment Outcomes and Responses

Nasal Polyp Score (NPS):

In the anti-IL-4 group, NPS decreased significantly from a baseline of 4.22 to 1.08 at <6 months and 0.19 at 6–12 months (both *p* < 0.0001 compared to baseline). This reduction was sustained across all follow-up intervals, with scores remaining significantly lower than baseline at 12–24 months (0.44, *p* < 0.0001), 24–36 months (0.00, *p* < 0.0001), and 36+ months (1.20, *p* = 0.0005).

**Figure 1:**
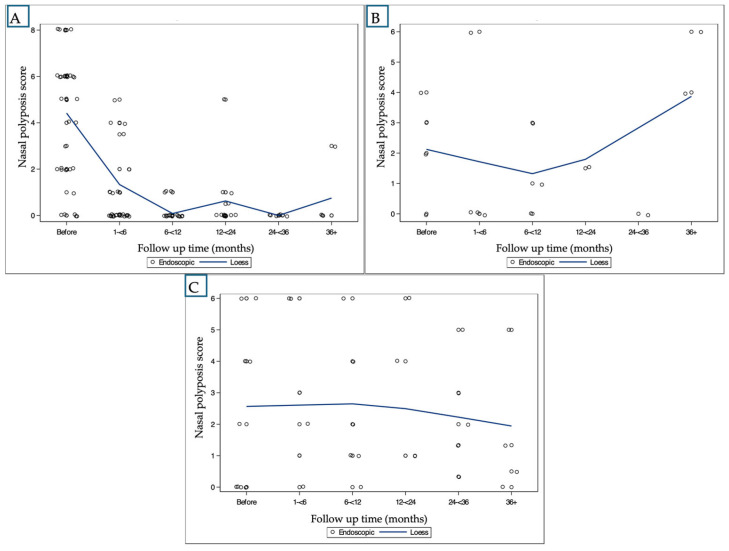
Longitudinal changes in NPS by biologic medication (LOESS smoothing): (**A**): patient treated with anti-IL-4; (**B**): patient treated with anti-IgE; (**C**): patient treated with anti-IL-5.

In the anti-IL-5 group, there was a trend toward NPS reduction over time, though it did not reach statistical significance (all *p* > 0.6 compared to baseline). In the anti-IgE group, no consistent trend was observed, and an increase in NPS was noted at 36+ months. The overall change is presented in Figure 1.

SNOT-22 Scores:

Patients receiving anti-IL-4 showed substantial reductions in SNOT-22 scores. A significant small improvement was seen at 12–24 and 24–36 months (−7.57, −5.57, respectively).

**Figure 2:**
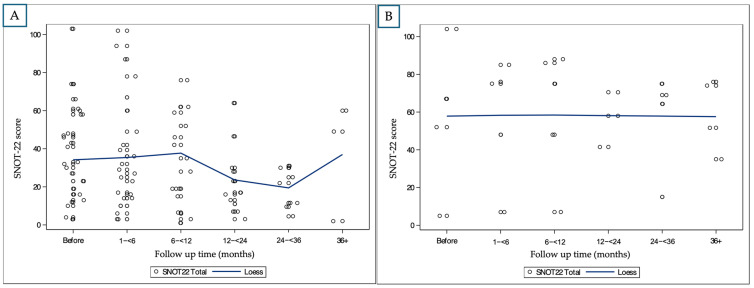
Longitudinal changes in SNOT-22 scores by biologic medication (LOESS smoothing): (**A**): patient treated with anti-IL-4; (**B**): patient treated with anti-IL-5. Note that patients receiving anti-IgE are very few; therefore, statistical analysis could not be applied.

In the anti-IgE and anti-IL-5 groups, SNOT-22 scores did not show consistent improvement, and in several intervals, a worsening of symptoms was observed (e.g., +4.0 for anti-IgE at 6–12 months, +3.8 for anti-IL-5 at 24–36 months). The overall change is presented in Figure 2.

**Figure 3:**
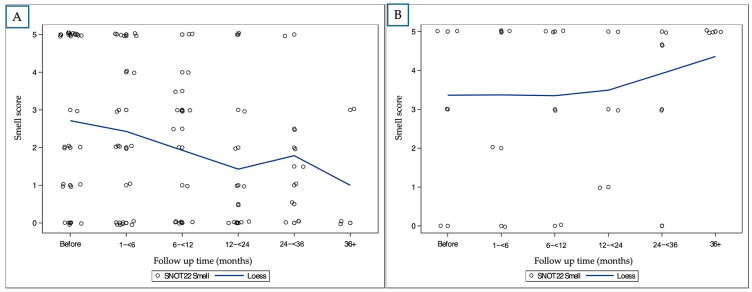
Longitudinal changes in sense of smell scores by biologic medication (LOESS smoothing): (**A**): patient treated with anti-IL-4; (**B**): patient treated with anti-IL-5. Note that patients receiving anti-IgE are very few; therefore, statistical analysis could not be applied.

Smell Scores:

Mean smell scores in the anti-IL-4 group improved steadily from a baseline of 2.96 to 1.13 at 12–24 months, with maintained improvement at later time points (1.29 at 24–36 months, 1.50 at 36+ months). The overall change is presented in Figure 3.

No meaningful improvement in smell scores was observed in the anti-IgE or anti-IL-5 groups. Scores remained unchanged or worsened over time, including a score of 5.0 at both baseline and 36+ months in the anti-IgE group. The relatively small sample sizes in both the anti-IgE and anti-IL-5 groups limit the strength of these findings.

Table 2 provides a summary of changes in nasal polyp score, SNOT-22, and smell function across the follow-up intervals for each biological treatment.

Treatment success was measured according to criteria outlined in the European Position Paper on Rhinosinusitis and Nasal Polyps (EPOS) by EPOS/EUFOREA 2023.

Categories across the time intervals. Anti-IL-4 showed a high proportion of excellent responders in the early and mid-follow-up periods, with over 75% achieving excellent response at 0–6 months and over 80% at 6–24 months. A more even distribution among poor, moderate, and excellent responses was observed at 36+ months.

**Figure 4:**
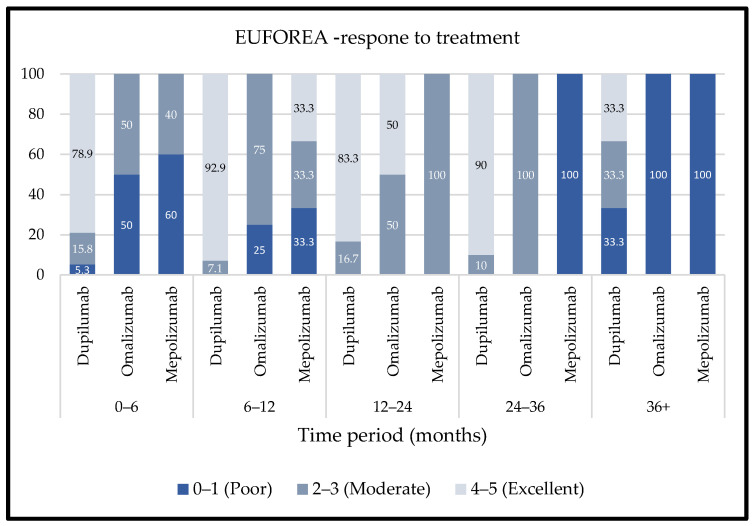
EUFOREA response to treatment.

Anti-IgE was associated with a higher proportion of poor responders across all time points, including 100% at 36+ months. Anti-IL-5 showed moderate response rates initially, with a decrease in excellent responders and an increase in poor responders over time (Figure 4).

For patients treated with anti-IL-4, SNOT-22 subdomains were analyzed according to Khan’s five-domain model (Table 3): rhinologic symptoms (Domain 1), extranasal rhinologic symptoms (Domain 2), ear/facial symptoms (Domain 3), sleep dysfunction (Domain 4), and psychological dysfunction (Domain 5).

A general trend of score reduction was observed across most domains, particularly between 12 and 36 months. Domain 1 (rhinologic symptoms) decreased significantly from 14.78 at baseline to 8.69 at 12–24 months (*p* = 0.031) and to 7.29 at 24–36 months (*p* = 0.025). Domain 5 (psychological dysfunction) also showed a significant reduction from 7.04 at baseline to 2.88 at 12–24 months (*p* = 0.044), with continued improvement at 24–36 months (2.64, *p* = 0.125). Domains 2 and 4 demonstrated numerical reductions but did not reach statistical significance. Domain 3 (ear/facial symptoms) showed minimal change over time and remained nonsignificant at all intervals. At 36+ months, an increase in scores was observed across all domains, though none were statistically significant.

### 2.3. Increasing Injection Intervals for Anti-IL-4 Treatment

A subgroup of 13 patients, representing 34% of those receiving anti-IL-4 therapy, underwent a gradual extension of their injection intervals from the standard two-week regimen to intervals of three, four, or six weeks. Clinical outcomes remained stable despite the reduced dosing frequency. Across these extended intervals, no significant increase in NPS or SNOT-22 scores was observed. Similarly, smell scores remained comparable to those recorded during the standard dosing period.

Patients selected for interval extension had a longer follow-up duration and distinct baseline characteristics compared to those who remained on the standard regimen as shown in Table 4. The median baseline NPS was higher in the interval-extension group (6.00 vs. 4.00), while SNOT-22 scores were similar (43.00 vs. 43.50). These patients also had lower total IgE levels (median 51.60 vs. 250.0), a higher rate of allergic sensitization (92.3% vs. 56.0%), and a lower prevalence of Samter’s triad (15.4% vs. 36.0%). Selection for interval extension was based on the treating physician’s (RE) subjective clinical judgment, in cases demonstrating stable disease control and favorable response.

### 2.4. Change in Treatment and Need for Additional Treatment

In our cohort, several patients changed their biologic therapy during follow-up due to insufficient response, based on either objective measurements (NPS) or subjective assessments (SNOT-22).

Four patients were switched from anti-IL-5 to anti-IL-4; three showed clinical improvement, while one patient, who did not improve, reverted to anti-IL-5. Two patients transitioned from anti-IgE to anti-IL-4, one demonstrated improvement in both NPS and SNOT-22, while the other improved in NPS but not in SNOT-22. One patient switched from anti-IL-4 to anti-IL-5 due to uncontrolled asthma.

Additionally, one patient is currently receiving dual therapy with anti-IgE and anti-IL-4 to optimize control of both asthma and nasal polyposis.

During the follow-up period, 9 patients (17.30%) underwent ESS, and 15 patients (28.84%) received systemic corticosteroids over 24 single visits. Systemic steroids were most often used during exacerbations of sinonasal symptoms related to infections. ESS was performed in cases where residual polyps or insufficient sinus drainage were observed during follow-up evaluations.

### 2.5. Discontinuation of Treatment and Side Effects

During each follow-up visit, patients were screened for potential side effects related to the biological therapy. Of all patients who attended follow-up visits, 43 (82.69%) reported no adverse effects. All the reported side effects occurred in patients receiving anti-IL-4. They included foul-smelling urine, joint pain, mild swelling at the injection site, skin erythema, eye itching, headache, abdominal pain, and elevated eosinophil counts. Most side effects were mild and reported during a single visit only.

Two patients discontinued treatment due to adverse effects: one due to significant eosinophilia and the other due to headache and abdominal pain, which occurred a few hours after injection and were subjectively intolerable.

## 3. Discussion

The short-term efficacy and safety of biologic therapy for CRSwNP has been well documented in the literature [6,8,10,11,12]. However, data on the long-term effects across all biological treatment classes remain limited [13]. In this study, we aimed to contribute additional real-world evidence regarding the long-term outcomes of various biological treatments for CRSwNP, with an additional focus on the quality of life, as assessed by the SNOT-22 and its subdomains. We also report on an emerging clinical practice of extending the dosing interval of dupilumab injections while maintaining excellent clinical results.

This cohort included 52 patients followed for up to five years. All patients included in this study met the EPOS/EUFOREA criteria for biologic eligibility [9,13]. Overall, a significant and sustained improvement in symptoms was observed in this real-world setting, with few adverse effects, most of which were mild and well tolerated.

In our cohort, anti-IL-4 was associated with sustained improvement across objective and subjective outcome measures. NPS decreased significantly from baseline and remained low through 36 months, with a slight increase thereafter (Figure 1A). SNOT-22 scores showed a marked reduction during the first two years, with a smaller but persistent effect observed beyond 36 months (Figure 2A). Smell function improved during the first two years and remained relatively stable in later follow-up visits.

Anti-IgE and anti-IL-5 showed more variable trends. Improvements in SNOT-22 and NPS were observed in early follow-up for both but were less consistent over time. Smell scores in these groups remained unchanged or worsened slightly.

The EPOS/EUFOREA response criteria [13] evaluate biologic treatment success based on five clinical domains: reduction in nasal polyp size, reduced need for systemic corticosteroids, improved quality of life, improved sense of smell, and reduced impact of comorbidities. Anti-IL-4 therapy demonstrated the highest proportion of excellent responders, particularly within the first three years of follow-up. An excellent response, defined as improvement in 4–5 of these domains, was achieved by over 75% of anti-IL-4-treated patients during the initial follow-up intervals, with this trend remaining relatively stable up to 24–36 months. Beyond 36 months, the distribution shifted, with a more even spread across poor (0–1 domains), moderate (2–3), and excellent response categories.

In contrast, anti-IgE and anti-IL-5 therapies showed a less favorable response distribution, with a higher proportion of patients meeting only 0–1 of the EUFOREA criteria, a trend that became more prominent after 24 months. These findings highlight differences in long-term response durability among biologic classes when assessed through a multidimensional, clinically relevant framework.

Analysis of the SNOT-22 subdomains among patients treated with dupilumab (anti–IL-4) according to the Khan model [14] demonstrated that the most prominent and sustained improvements occurred in the nasal and emotional domains. These findings suggest that while dupilumab provides broad symptom relief, its most consistent effects are observed in areas directly related to nasal inflammation and the emotional burden of chronic disease. This is particularly relevant given the growing evidence that chronic rhinosinusitis, including CRSwNP, is associated with an increased risk of depression and anxiety. A nationwide cohort study found that patients with CRS have a significantly higher risk of developing these mental health conditions compared to individuals without CRS [15]. These findings underscore the bidirectional relationship between psychological stress and nasal polyposis, where emotional distress may exacerbate disease activity, and conversely, the burden of CRS may contribute to psychological symptoms. Taken together, these insights highlight the multidimensional impact of biologic therapy and the importance of evaluating quality of life not only globally, but also across specific symptom domains [16,17].

Our finding, of the clinically favorability of dupilumab (anti-IL-4), aligns with previously published literature. In a meta-analysis by Wu et al., which compared dupilumab, omalizumab, and mepolizumab for the treatment of CRSwNP, dupilumab ranked highest in terms of efficacy, based on improvements in NPS, and safety, with the lowest incidence of adverse events. As for anti-IL-5 and anti-IgE therapies, it is difficult to draw definitive comparisons within our cohort due to the small number of patients in each treatment group. However, in the same meta-analysis by Wu et al., omalizumab was identified as the second-best option overall, while mepolizumab, although demonstrating comparable efficacy, was associated with the highest rate of adverse events [7].

In our cohort, improvement in nasal polyp size consistently came together with improvement in the sense of smell, and conversely, worsening in NPS was accompanied by olfactory decline. This parallel trend aligns with the accepted hypothesis that conductive olfactory loss in CRSwNP is primarily driven by an obstructive component, where mechanical blockage of the olfactory cleft limit odorant access [18].

Switching between biologics occurred in cases of intolerable side effects or inadequate response to initial therapy, often without a washout. Although this approach was generally well tolerated, it should be carefully discussed with patients beforehand to minimize the risk of unnecessary switching or the need to revert to previous treatment. The change response data in our cohort are not presented in this paper.

It is noteworthy that in one case, an interdisciplinary approach led to the use of dual biologic therapy: dupilumab (anti-IL-4) was prescribed for CRSwNP, and omalizumab (anti-IgE) was prescribed due to severe persistent asthma. Although both omalizumab [6] and dupilumab [19] are individually approved for the treatment of CRSwNP and asthma, respectively, the patient was unable to maintain symptom control when treated with only one of the two agents. Data on dual biological therapy remain limited and are mostly restricted to case reports. Further studies are warranted to evaluate the safety, efficacy, and cost-effectiveness of this approach.

Dupilumab, although highly effective, is associated with considerable long-term costs. A cost–utility analysis reported that dupilumab yields 8.95 quality-adjusted life years (QALYs), a measure that combines both length and quality of life, at a total 10-year cost exceeding USD 530,000, compared to 9.8 QALYs and approximately USD 50,000 for ESS [20,21,22]. Tapering, or extending the dosing intervals, is a widely adopted strategy in the use of biologics for chronic inflammatory diseases [23,24], aimed at reducing treatment burden, minimizing side effects, and improving cost-effectiveness. The first evidence from the LIBERTY NP SINUS-52 trial showed that patients transitioning from bi-weekly to monthly dupilumab injections maintained comparable clinical outcomes [25]. The real-world application of this approach in CRSwNP is in its early stage of investigation. To the best of our knowledge, no published data currently characterize which patients are most likely to benefit from or be eligible for injection interval extension.

Frankenberger et al. described a cohort of patients who independently extended their injection intervals after achieving satisfactory therapeutic outcomes; however, the study did not report on the characteristics of these patients or what distinguished them from those who did not prolong treatment intervals [16]. Similarly, Lans et al. presented real-world data on tapering dupilumab injections as infrequently as every 12 weeks, while maintaining clinical effectiveness. Yet, this study also lacked an analysis of which patient profiles were suitable for tapering and which failed to sustain the response [26]. Most recently, Yoon et al. published a retrospective analysis of 44 CRSwNP patients treated with dupilumab for more than six months, evaluating the impact of interval adjustment based on SNOT-22 scores. Patients were stratified by baseline SNOT-22, with treatment goals targeting either a score below 20 or a ≥50% improvement. Injection intervals reached up to 12 weeks in some patients. Patient satisfaction remained consistently high, and improvement in nasal symptoms significantly correlated with greater satisfaction [27].

In our cohort, a favorable response to dupilumab (anti–IL-4) therapy led us in approximately one-third of patients to extend the injection intervals, and, in some cases, to reduce the use of local nasal treatments. Patients selected for interval extension were chosen at the discretion of the treating physician (RE), based on documented clinical stability across visits, absence of exacerbations, and good patient-reported outcomes. This personalized, response-driven approach is increasingly being explored and may offer a pathway to optimize both treatment efficacy and long-term economic sustainability, particularly in patients with stable disease and milder inflammatory profiles. However, the need to stratify and identify suitable candidates for tapering remains a key clinical challenge.

This study has several limitations. First, it is retrospective, with a relatively small sample size derived from a single tertiary center, in which all patients are being evaluated by the same clinical team. While this may contribute to consistency in assessment, it limits generalizability.

Second, although the cohort includes patients treated with various biologic agents, the majority received anti-IL-4 therapy, limiting the ability to draw meaningful comparisons across treatment groups, particularly for those represented by only a small number of patients.

Additionally, follow-up intervals were not uniform across patients. Although statistical methods were employed to address this variability, an inherent bias remains patients who returned for more frequent or longer-term follow-up may differ systematically from those with sparse or discontinued follow-up, potentially introducing selection and information bias.

This study was not powered or designed for formal hypothesis testing. As a retrospective, observational cohort with relatively small subgroups, particularly in the anti-IL-5 and anti-IgE arms, many statistical comparisons were limited to descriptive analyses. While *p*-values for key outcome measures, such as nasal polyp score and SNOT-22, were appropriate, effect sizes and confidence intervals were not consistently calculable due to variability in sample size and follow-up. Nonetheless, the strength of our data lies in the long-term, single-institute real-world perspective it offers, including repeated measures across multiple time points and clinically relevant endpoints.

Future work is needed in a few areas: While the EPOS/EUFOREA criteria [13] provide a useful framework for assessing biologic treatment response in CRSwNP, their current form remains relatively vague and lacks specific thresholds for improvement, especially in the long-term context. For example, the extent of reduction required in nasal polyp size, the minimal clinically important difference in quality-of-life scores (e.g., SNOT-22), or the degree of olfactory improvement necessary to classify a “positive response” are not clearly defined or standardized based on time intervals. The standardization of those parameters would improve consistency in outcome reporting and facilitate better comparisons across studies and patient populations.

More research is needed to evaluate the long-term effects of biological therapy and to conduct double-blind, standardized trials comparing anti-IL-4, anti-IgE, and anti-IL-5 therapies for CRSwNP patients. Such studies may help identify which patient subpopulations derive the greatest benefit from each agent.

In addition, further data are needed to clarify the clinical effects of injection interval extension, specifically, when it is appropriate to initiate tapering, what are the optimal and maximal intervals, which patients are best suited for this approach, and what is its potential impact on treatment costs and overall healthcare resource utilization.

## 4. Materials and Methods

The study is a retrospective observational study involving patients who received biological treatment for CRSwNP from 2019 to 2024 at a tertiary medical center. The study was approved by the local Helsinki Committee [0361-22-HMO].

All patients ≥ 18 suffered from CRSwNP and met the EPOS 2020 criteria for being eligible for biological treatment due to severe disease resistant to intranasal corticosteroids (INCS), systemic corticosteroids, and ESS [1]. As all biologics are approved as an add-on therapy to topical nasal steroids, all patients were on long-term INCS.

All patients had evidence of Type 2 inflammation based on either laboratory and pathological results, or clinical findings (allergy with a positive skin prick test; presence of late-onset asthma; NSAIDs intolerance). Follow-up visits were on irregular timespan intervals.

Data on the patient’s status before imitation of treatment and on every follow-up visit were retrieved from the patient’s electronic medical records. This included demographics, clinical and background features, nasal polyp score (NPS), ranging from 0 to 8, SNOT-22 questionnaire score, ranging from 0 to 110, subjective sense of smell, ranging from 0 to 5, and laboratory data when available. For a more detailed description of the measured data, the reader is referred to our previous study [12]. Additionally, we recorded the occurrence of adverse events, the need for ESS, the use of systemic corticosteroids during therapy, and the injection intervals at the time of the last follow-up visit. Treatment success was measured according to criteria outlined in the European Position Paper on Rhinosinusitis and Nasal Polyps (EPOS) by EPOS/EUFOREA 2023 [13].

Statistical analysis was generated using SAS Software, Version 9.4, SAS Institute Inc., Cary, NC, USA. Continuous variables were presented by Mean ± Std or Median and IQR. Categorical variables were presented by (N, %).

Visit times were categorized as the last visit before treatment, less than 6 months, 6 to 12 months, 12 to 24 months, 24 to 36 months, and 36 months or more. For each of these intervals, mean values of study parameters were calculated and used in subsequent analysis.

LOESS smoothing [28] was used to plot study parameters vs. follow-up time intervals. To check the effect of follow-up time on study parameters, mixed models with repeated measures, for multiple visits of the same subject, were used. Follow-up time was fitted as a categorical variable, since LOESS plots indicated non-linear trends. The Tukey–Kramer method was used to correct for multiple comparisons. *p*-Values below 0.05 were considered significant.

## 5. Conclusions

In conclusion, this real-world study reinforces the long-term effectiveness and safety of biologic therapy for patients with CRSwNP, particularly with anti-IL-4 treatment. Sustained improvements were observed in nasal polyp size, quality of life, and olfaction, with the most pronounced effects seen in the nasal and emotional symptom domains. The EPOS/EEUFOREA criteria proved valuable in assessing treatment response over time, though standardization of domain-specific thresholds is needed. Extending injection intervals in selected patients shows promise for maintaining disease control while potentially reducing treatment burden and cost. As biologics become more widely used, further high-quality studies are needed to determine optimal treatment duration, tapering down strategies, and to better define which patients benefit most from each biologic agent.

## Figures and Tables

**Table 1:** Patients’ baseline characteristics.

Patient Characteristics	Baseline			
	All	Anti-IL-4	Anti-IgE	Anti-IL-5
Number of patients	52	38	4	10
Age *	54.14 (43.58–65.49, 50)	57.68 (43.58–66.65, 37)	42.21 (36.47–51.39, 4)	52.10 (43.69–60.95, 9)
Gender (female) **	19 (36.54)	13 (34.21)	1 (25)	5 (50)
Samter’s triad **	15 (28.85)	11 (28.95)	1 (25)	3 (30)
Asthma **	46 (88.46)	33 (86.84)	3 (75)	10 (100)
Allergic status **	35 (67.31)	26 (68.42)	3 (75)	6 (60)
Blood Eosinophils% *	7.4 (4.2–11.2, 33)	6.65 (3.1–9.15, 24)	20 (4.2–23, 3)	9.85 (7.2–12.4, 6)
Total IgE *	130 (40.6–274.5, 24)	191.5 (40.6–335.5, 16)	177 (114–274, 4)	51.65 (18.8–150.5, 4)
Number of prior ESS *	2 (1–2, 51)	2 (1–2, 37)	1.5 (1–2, 4)	1.5 (1–3, 10)
Time from ESS * (months)	20 (5–27, 46)	21 (5–32, 34)	13 (5.5–22.5, 4)	19.5 (9–29, 8)
Nasal polyposis score *	4 (2–6, 49)	5 (2–6, 36)	2.5 (1–3.5, 4)	2 (0–4, 9)
SNOT-22 score *	43 (19–60, 33)	33 (16–58, 27)	62 (53–71, 2)	59.5 (28.5–85.5, 4)
Follow-up time ***	19.13 (20.45)	14.18 (13.89)	43.00 (47.69)	28.40 (19.13)

* Median (Q1–Q3, N); ** N (%); *** Mean (SD).

**Table 2:** Clinical measurements during treatment.

Treatment	Time (Months)	NPS *	SNOT-22 **	Smell *
Anti-IL-4	Baseline	4.22, 36	38.52, 27	2.96, 27
	<6	1.08, 36 ***	+2.00, 20	2.44, 27
	6–12	0.19, 21 ***	−4.00, 16	2.05, 20
	12–24	0.44, 18 ***	−7.57, 14 ***	1.13, 16 ***
	24–36	0.0, 15 ***	−5.57, 14 ***	1.29, 14 ***
	36+	1.20, 5 ***	−5.00, 2	1.50, 4
Anti-IgE	Baseline	2.25, 4	62.0, 2	5.00, 2
	<6	1.50, 4	−3.50, 2	-
	6–12	1.75, 4	+4.00, 1	5.00, 1
	12–24	1.50, 4	–	-
	24–36	0.0, 1	–	-
	36+	4.46, 13	–	4.70, 10
Anti-IL-5	Baseline	2.44, 9	57.00, 4	3.25, 4
	<6	3.00, 6	11.33, 3	3.40, 5
	6–12	1.88, 8	−2.67, 3	4.00, 7
	12–24	2.57, 7	+3.50, 2	3.00, 6
	24–36	1.67, 9	+10.20, 5	3.50, 8
	36+	1.43, 7	+24.00, 1	4.73, 11

* Mean, N; ** mean change from baseline, N; *** *p*-value < 0.05.

**Table 3:** SNOT-22 subdomain during treatment.

Domain	1	2	3	4	5
Baseline	14.78, 27	5.74, 27	4.07, 27	8.81, 27	7.04, 27
<6	12.70, 33	5.39, 33	4.52, 33	8.82, 33	7.24, 33
6–12	11.23, 22	5.41, 22	3.36, 22	7.86, 221	4.95, 22
12–24	8.69, 16 ***	4.38, 16	2.69, 16	5.44, 16 ***	2.88, 16 ***
24–36	7.29, 14 ***	2.50, 14 ***	1.64, 144	4.07, 14	2.64, 14
36+	11.25, 4	5.75, 46	9, 4	9.25, 4	9.75, 4

*** *p*-value < 0.05.

**Table 4:** Anti-IL-4 patients’ baseline characteristics.

Patient Characteristics	Baseline	
	Regular Interval	Extended Intervals
Number of patients	25	13
Age *	56.93 (43.92–64.34)	62.80 (40.18–71.05)
Gender (female) **	32.0%	38.5%
Samter’s triad **	36.0%	15.4%
Asthma **	88.0%	84.6%
Allergic status **	56.0%	92.3%
Blood Eosinophils% *	6.90 (1.50–9.15)	6.35 (4.15–9.30)
Total IgE *	250.0 (52.0–760.0)	51.60 (29.60–243.00)
Number of prior ESS *	2.00 (1.00–2.00)	2.00 (1.00–3.00)
Time from ESS * (months)	18.00 (4.00–26.00)	23.00 (19.00–54.00)
Nasal polyposis score *	4.00 (2.00–6.00)	6.00 (4.00–6.00)
SNOT-22 score *	43.50 (31.50–69.50)	43.00 (29.00–62.00)

* Median (Q1–Q3, N); ** N (%).

## Data Availability

Data will be shared per request.

## Abbreviations

CRS	Chronic Rhinosinusitis
CRSwNP	Chronic Rhinosinusitis with Nasal Polyps
CRSsNP	Chronic Rhinosinusitis without Nasal Polyps
ESS	Endoscopic Sinus Surgery
INCS	Intranasal Corticosteroids
NSAIDs	Non-Steroidal Anti-Inflammatory Drugs
IL	Interleukin
IgE	Immunoglobulin E
NPS	Nasal Polyp Score
QALY	Quality-Adjusted Life Year
RCTs	Randomized Controlled Trials
EPOS	European Position Paper on Rhinosinusitis and Nasal Polyps
EUFOREA	European Forum for Research and Education in Allergy and Airway Diseases
SNOT-22	22-item Sino-Nasal Outcome Test
